# Immunosuppressive and angiogenic cytokine profile associated with *Bartonella bacilliformis* infection in post-outbreak and endemic areas of Carrion's disease in Peru

**DOI:** 10.1371/journal.pntd.0005684

**Published:** 2017-06-19

**Authors:** Maria J. Pons, Cláudia Gomes, Ruth Aguilar, Diana Barrios, Miguel Angel Aguilar-Luis, Joaquim Ruiz, Carlota Dobaño, Juana del Valle-Mendoza, Gemma Moncunill

**Affiliations:** 1 Centro de Investigación e Innovación de la Facultad de Ciencias de la Salud de la Universidad Peruana de Ciencias Aplicadas, Lima, Perú; 2 Instituto de Investigación Nutricional, Lima, Perú; 3 ISGlobal, Barcelona Centre for International Health Research, Hospital Clínic - Universitat de Barcelona, Catalonia, Spain; University of Connecticut Health Center, UNITED STATES

## Abstract

Analysis of immune responses in *Bartonella bacilliformis* carriers are needed to understand acquisition of immunity to Carrion’s disease and may allow identifying biomarkers associated with bacterial infection and disease phases. Serum samples from 144 healthy subjects from 5 villages in the North of Peru collected in 2014 were analyzed. Four villages had a Carrion’s disease outbreak in 2013, and the other is a traditionally endemic area. Thirty cytokines, chemokines and growth factors were determined in sera by fluorescent bead-based quantitative suspension array technology, and analyzed in relation to available data on bacteremia quantified by RT-PCR, and IgM and IgG levels measured by ELISA against *B*. *bacilliformis* lysates. The presence of bacteremia was associated with low concentrations of HGF (p = 0.005), IL-15 (p = 0.002), IL-6 (p = 0.05), IP-10 (p = 0.008), MIG (p = 0.03) and MIP-1α (p = 0.03). In multi-marker analysis, the same and further T_H_1-related and pro-inflammatory biomarkers were inversely associated with infection, whereas angiogenic chemokines and IL-10 were positively associated. Only EGF and eotaxin showed a moderate positive correlation with bacteremia. IgM seropositivity, which reflects a recent acute infection, was associated with lower levels of eotaxin (p = 0.05), IL-6 (p = 0.001), and VEGF (p = 0.03). Only GM-CSF and IL-10 concentrations were positively associated with higher levels of IgM (p = 0.01 and p = 0.007). Additionally, IgG seropositivity and levels were associated with high levels of angiogenic markers VEGF (p = 0.047) and eotaxin (p = 0.006), respectively. Our findings suggest that *B*. *bacilliformis* infection causes immunosuppression, led in part by overproduction of IL-10. This immunosuppression probably contributes to the chronicity of asymptomatic infections favoring *B*. *bacilliformis* persistence in the host, allowing the subsequent transmission to the vector. In addition, angiogenic markers associated with bacteremia and IgG levels may be related to the induction of endothelial cell proliferation in cutaneous lesions during chronic infections, being possible candidate biomarkers of asymptomatic infections.

## Author summary

Carrion’s disease is a neglected vector-borne disease limited to vulnerable population of Ecuador, Colombia and specially Peru. This illness consist in two distinct phases, the Oroya fever and Peruvian wart, but exist a high percentage of asymptomatic carriers in endemic areas that should be detected in order to perform correct surveillance and control. Moreover, information on immunity and immune responses to *Bartonella bacilliformis*, the causative agent, is very limited and represents a challenge. This study identified serum biomarkers associated with Carrion’s disease asymptomatic infections. In addition, it provides novel information on the complex host-immune interactions in these individuals, suggesting that the bacteria induces an immunosuppression in the acute phase that is maintained in later phases with low levels of bacteremia. This immunoppression would help the establishment and persistence of the infection.

## Introduction

Carrion’s disease (CD) (ORPHANET 64692) is a tropical, neglected poorest-linked illness, endemic in low-income areas of Peru, but also affecting specific areas of Ecuador and Colombia, with sporadic cases reported in Bolivia and Chile [1]. It is estimated that approximately 1.7 million of South Americans are at risk of CD [1–3]. The bacteria *Bartonella bacilliformis* is the etiological agent of CD, but recently other *Bartonella* spp. have been related to this illness [4–6]. In the human host, *B*. *bacilliformis* is an intracellular pathogen that invades mainly erythrocytes and vascular endothelial cells [7]. *B*. *bacilliformis* is transmitted by the bite of sand flies (members of the genus *Lutzomyia*) and no reservoir has been identified other than humans, making it an eradicable disease [1,8]. Nowadays, CD is located in a restricted area, but in this era of globalization a future expansion to other areas cannot be ruled out, as has been described for other neglected diseases [8]. Unfortunately, no rapid diagnostic method to detect *B*. *bacilliformis* and CD has yet been developed to be available for endemic areas [8]. Currently, the infection is diagnosed by blood smear but this has several limitations including low sensitivity [9–10] and diagnosis error [11].

CD is clinically characterized by two phases. The first one, named Oroya’s Fever, consists in the acute infection that mainly affects young children (>60% of cases) and is characterized by fever, acute bacteremia and severe hemolytic anemia [12,13]. In absence of adequate treatment, Oroya's Fever achieves high levels of mortality (44% to 88%) due to high bacteremia and opportunistic infections [3]. Complications during the acute phase and secondary infections are common, likely due to transient immunosuppression. The second phase, known as “Peruvian wart”, is a chronic phase usually occurring weeks or months after the acute phase and leads to a series of cutaneous lesions due to the bacterial induction of endothelial cell proliferation [3,12]. In addition, asymptomatic infections of undefined duration are common in people from endemic areas [14], with a case of asymptomatic bacteremia of up to 3 years reported [15]. Estimates of the real burden of asymptomatic cases may not be accurate, but, we have recently reported rates of 37% carriers in post-outbreak areas and 52% in an endemic area by real time Polymerase Chain Reaction (RT-PCR) [16]. These symptomless infections that go unnoticed are probably the major reservoir of *B*. *bacilliformis*, and allow the transmission of the bacteria. Therefore, efforts leading to the development and application of new more efficient diagnostic techniques that can be used in endemic field areas are required to detect and distinguish acute, chronic and asymptomatic infections, in order to control and even eradicate CD.

Information on immunity to CD and immune responses to *B*. *bacilliformis* is very limited and represents a challenge, due to the neglect of the disease and difficulty to obtain samples from the remote areas affected. Both humoral and cellular immune responses are induced during acute infection of CD [3]. It seems that antibody immunity to *B*. *bacilliformis* infection build up with age and exposure is lifelong, although it probably confers only partial protection and seropositive individuals may be asymptomatic carriers or have Peruvian warts [3]. Leukocytosis and anemia are probably responsible of the immunosuppression associated with acute infections, but cellular immune mechanisms involved remain unknown [3]. To our knowledge it is not known either what happens in asymptomatic subjects, in whom the infection persists [3].

In this exploratory study, we measured cytokines, chemokines and growth factors by quantitative multiplex fluorescent bead-based suspension arrays with the aim of providing information on the immune response to *B*. *bacilliformis* and identifying potential serum biomarkers of *B*. *bacilliformis* infection in non-acute individuals. The technology used allows evaluating simultaneously a high number of analytes using low sample volumes, which facilitates to extend the studies to specially CD vulnerable young populations.

## Material and methods

### Geographical area

A cross-sectional survey was done in 5 villages of Piura (northern Peru) on March 2014 [16]. In 4 of them (Guayaquiles, Los Ranchos, Mayland, and Tunal) an Oroya fever outbreak was reported between November 2013 and March 2014, while Huancabamba is a well-established endemic area for this illness [16–17]. In Guayaquiles, Los Ranchos, Mayland and Tunal, study participants recruited were volunteers diagnosed with CD (by clinical symptoms and/or thin blood smear) during the previous outbreak. All subjects received ciprofloxacin antibiotic treatment during 14 days following diagnosis according to national guidelines. In Huancabamba, the volunteers were randomly recruited by house-to-house visits [16]. Clinical and demographical data were recorded [16].

### Ethics statement

The study was approved by the Universidad Peruana de Ciencias Aplicadas Ethics Committee and the Hospital Clínic of Barcelona Ethics Committee. Written informed consent was obtained from all adults and parents or guardians of any child participant on their behalf before recruitment.

### Sampling

Serum samples from a total of 144 individuals out of 177 were randomly selected. Sample size was limited by the number of tests that could be performed in two Luminex kit plates. In a previous study, IgG and IgM levels against *B*. *bacilliformis* lysate were measured by ELISA, and *B*. *bacilliformis* was detected and quantified by RT-PCR [16].

### Quantification of cytokines, chemokines and growth factors

The Cytokine Human Magnetic 30-Plex Panel from Life Technologies was used to measure the concentrations (pg/mL) of the following cytokines, chemokines and growth factors in serum: epidermal growth factor (EGF), eotaxin, fibroblast growth factor (FGF), granulocyte colony-stimulating factor (G-CSF), granulocyte macrophage colony-stimulating factor (GM-CSF), hepatocyte growth factor (HGF), Interferon (IFN)-α, IFN-γ, interleukin (IL)-1RA, IL-1β, IL-2, IL-2R, IL-4, IL-5, IL-6, IL-7, IL-8, IL-10, IL-12 (p40/ p70), IL-13, IL-15, IL-17, IP-10, monocyte chemoattractant protein-1 (MCP-1), monokine induced by gamma interferon (MIG), macrophage inflammatory protein (MIP)-1α, MIP-1β, RANTES, tumor necrosis factor (TNF), and vascular endothelial growth factor (VEGF). Fifty μL of all samples were tested in single replicates distributed in two plates following manufacturer’s instructions. Each plate included 16 2-fold serial dilutions in single replicates (at the exception of the highest concentration that was duplicated) of a standard sample provided by the vendor with known concentration of each analyte. Two blank controls and three positive controls in duplicate of high, medium and low concentrations prepared from a reference sample were also included in each plate for quality assurance and quality control purposes. Samples were acquired on a Luminex 100/200 instrument and analyzed in xPONENT software 3.1. The standard curves were fitted based on five-parameter log-logistic models. To account for background noise, median fluorescent intensity (MFI) of blank controls was subtracted to MFI of samples. The higher limit of quantification (HLOQ) was based on the higher dilution of the standard curve; and the lower limit of quantification (LLOQ) was calculated as the mean of blanks plus 2SD. When sample MFIs were out of quantification limits, an arbitrary value was imputed (half of the expected concentration of the LLOQ for values < LLOQ and twice the expected concentration of the HLOQ for values > HLOQ). FGF, IL-1β, IL-17 and IL-7 were discarded from the analysis because > 80% measurements were out of range. In addition, the transforming growth factor β (TGF-β) was evaluated by an ELISA commercial kit (LabClinics) following manufacturer’s instructions.

### Statistical analysis

The studied population was categorized into 5 age groups (≤ 10 years, 11–25 years, 26–55 years, 56–69 years and ≥ 70 years) as in our previous publication [16]. Localities were grouped to post-outbreak (Guayaquiles, Los Ranchos, Mayland, and Tunal) or endemic areas (Huancabamba). IgM and IgG seropositivities were defined according to Finite Mixture Models (FMM), with a cut off of 0.351 optical densities for IgM and 0.533 for IgG reported in Gomes C. *et al* [16]. Comparisons between groups for categorical variables were done using Fisher’s exact test. Demographic continuous variables were analyzed using the non-parametric Wilcoxon rank-sum test. IgG, IgM, marker concentration, bacteremia and age data were log_10_ transformed for further analysis. Comparison of levels of IgG and IgM by RT-PCR results were performed through t-test with welch correction. The effect of RT-PCR results, antibody responses, area and age on marker levels were assessed through separate simple linear regressions for each marker, with marker concentration as outcome and RT-PCR results, IgG responses, IgM responses, age and area as the predictor variable. The effect of RT-PCR results and antibody responses on single marker concentrations adjusting by age and area was assessed in multiple linear regressions with age and area as covariates and marker concentration as outcome. Correlations between continuous *B*. *bacilliformis* RT-PCR measurements, immunoglobulin levels, and cytokine, chemokine and growth factor concentrations were also calculated by Spearman correlation.

To identify clusters of markers simultaneously associated with RT-PCR positivity, we performed partial least square discriminant analysis (PLS-DA). PLS-DA allows compressing a high number of collinear variables into a new set of uncorrelated variables (components) that explain most of the variance of the data and also the outcome of interest (RT-PCR positivity in our case). The most predictive components were selected by logistic regressions based on P-values <0.05 with RT-PCR positivity as the outcome. In addition, we also performed a multivariable model with the three components as predictors and RT-PCR positivity as outcome. We considered that biomarkers substantially contributed to the components when had loadings > |0.3|.

All p-values were considered statistically significant when <0.05. P-values were adjusted for multiple testing to control the false discovery rate using the Benjamini-Hochberg approach (Supplementary information), but due to the exploratory character of the study results are discussed taking into account raw p-values and data were interpreted based on internal consistence, biological plausibility and previous literature. Box plots were performed using Graphpad Prism version 6.00 (GraphPad Software, San Diego California USA) and all data collected were analyzed using R software version 3.2.4 (2016-03-10) [18]. The DiscriMiner package [19] was used to perform the PLS-DA analysis, the ggplot2 package [20] was used to perform PLS-DA graphs and scatter plots. ROC analysis was conducted using an *auc* and *roc*.*area* functions from pROC package [21]. The reshape package was used for data manipulation purposes [22].

## Results

### Characteristics of studied samples

A total of 177 serum samples were collected in 2014 in 5 villages of Northern Peru [16]. Of these, 144 sample sera (Guayaquiles—23, Los Ranchos—39, Mayland—10, Tunal—51, and Huancabamba -21) were analyzed in this study. Demographic characteristics, RT-PCR data and IgM and IgG seroprevalences for the 177 subjects are described in our previous study [16]. Characteristics of the subset of 144 study subjects analyzed here were similar (Table 1), showing no bias in the sample selection for biomarker profiling. No significant differences were found in sex, age or area between subjects with detectable bacteremia (RT-PCR positives) and subjects without detectable bacteremia (RT-PCR negatives). Age distribution was significantly different in IgM seropositive compared to IgM seronegative individuals, with higher prevalence of younger individuals among the seropositive ones. The contrary tendency was observed for IgG seropositives and negatives and a higher frequency of IgG seropositives was found in the endemic area than IgG seronegatives. We have previously reported that around 32.9% and 26% of RT-PCR positive individuals were IgM and IgG seropositive against *B*. *bacilliformis* lysate, respectively [16]. In the subset of subjects analyzed in this study, IgM and IgG seroprevalences were of 33.3% and 25.9%, respectively and no significant differences in IgM and IgG seropositivity (p = 0.72 and p = 0.46, respectively) or IgM and IgG levels (p = 0.71 and p = 0.72, respectively) were detected according to RT-PCR results. Therefore, we consider IgM responses as markers of recent acute infection, and IgG responses as markers of previous exposure and immunity.

**Table 1:** Epidemiologic and demographic characteristics of study participants stratified by RT-PCR, IgM and IgG results.

	Samples	RT-PCR +	RT-PCR -	p-value	IgM+	IgM-	p-value	IgG+	IgG-	p-value
	(N = 144)	(N = 54, 37.5%)	(N = 90, 62.5%)		(N = 52, 36.1%)	(N = 92, 63.9%)		(N = 43, 29.9%)	(N = 101, 70.1%)	
**Sex**^a^				p = 0.296			p = 0.288			p = 0.062
Female	88 (61.1)	30 (55.6)	58 (64.4)		35 (67.3)	53 (57.6)		21 (48.8)	67 (66.3)	
Male	56 (38.9)	24 (44.4)	32 (35.5)		17 (32.7)	39 (42.4)		22 (51.2)	34 (33.7)	
**Age Median years (IQR)** ^b^^,^ ^c^	40 (18, 52)	36 (12.25,51)	40 (27, 56)	p = 0.114	27.5 (12, 44.75)	44 (31, 56.5)	p< 0.001	40 (32, 51.75)	39 (13, 54)	p = 0.351
**Age group N (%)**^a^^,^ ^c^				p = 0.125			p = 0.002			p = 0.068
<10	19 (13.3)	9 (16.7)	10 (11.2)		10 (19.2)	9 (9.9)		1 (2.4)	18 (17.8)	
11 to 25	22 (15.4)	13 (24.1)	9 (10.1)		14 (26.9)	8 (8.8)		6 (14.3)	16 (15.8)	
26 to 55	72 (50.4)	24 (44.4)	48 (53.9)		21 (40.4)	51 (56)		27 (64.3)	45 (44.6)	
56 to70	20 (14)	6 (11.1)	14 (15.7)		7 (13.5)	13 (14.3)		6 (14.3)	14 (13.9)	
>71	10 (7)	2 (3.7)	8 (9)		0 (0)	10 (11)		2 (4.8)	8 (7.9)	
**Area**^a^				0.337			0.624			p<0.001
Post-outbreak	123 (85.4)	44 (81.5)	79 (87.8)		43 (82.7)	80 (87)		29 (67.4)	94 (93.1)	
Endemic	21 (14.6)	10 (18.5)	11 (12.2)		9 (17.3)	12 (13)		14 (32.6)	7 (6.9)	

Abbreviations: IQR, interquartile range.

^a^ P-values were computed through two sided Fisher’s exact test.

^**b**^ P-values were computed through Wilcoxon rank-sum test.

^**c**^ Age is missing from one individual, who was RTPCR-, IgM- and IgG+.

### Effect of *B*. *bacilliformis* bacteremia on biomarker levels

**Fig 1:**
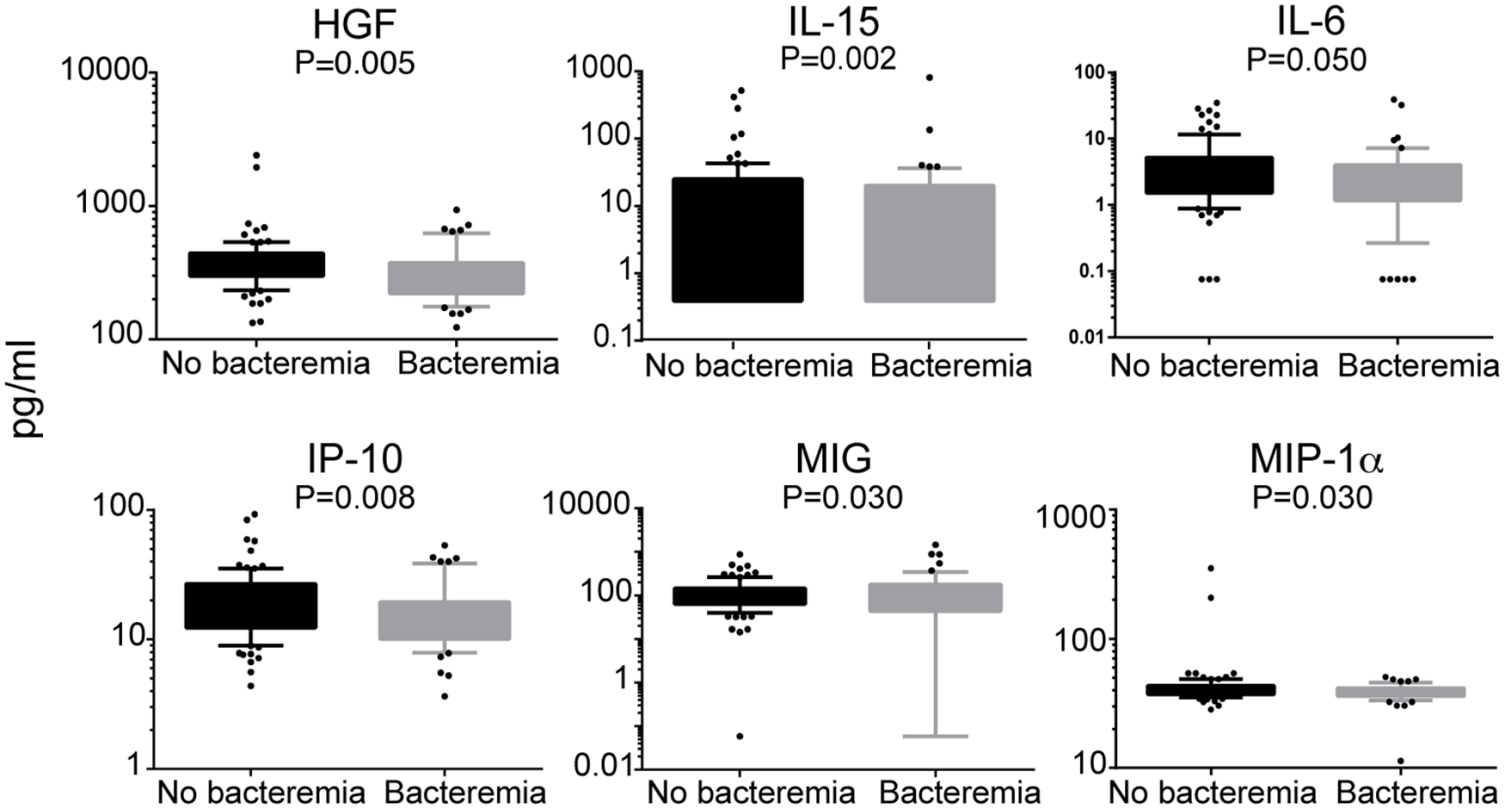
Serum biomarker concentrations in subjects with and without detectable *B*. *bacilliformis* bacteremia. Boxplots illustrate the 25^th^ and 75^th^ quartiles, and whiskers the percentile 10–90 of biomarkers significantly associated with RT-PCR positivity. P-values were computed through unadjusted linear regressions.

We examined the effect of *B*. *bacilliformis* bacteremia on biomarker concentrations. Subjects with detectable bacteremia had lower levels of HGF (p = 0.005), IL-15 (p = 0.002), IL-6 (p = 0.05), IP-10 (p = 0.008), MIG (p = 0.03) and MIP-1α (p = 0.03) (Fig 1, S1 Table) compared to subjects without bacteremia. IL-1RA also had a tendency to be lower in subjects without bacteremia (p = 0.059).

Age positively correlated with levels of eotaxin (p<0.001), IL-6 (p = 0.008) and MCP-1 (p = 0.002), and negatively with IL-12 (p<0.001) and IL-2 (p = 0.026) (S1 Table). Study area was also associated with levels of some markers, with higher levels of EGF (p<0.001), eotaxin (p = 0.066), and TNF (p = 0.032) and lower levels of IP-10 (p = 0.019) in the endemic area compared to the post-outbreak area (S1 Table). Therefore, we adjusted the analysis of the effect of RT-PCR positivity on biomarker levels by age and area (S2 Table). HGF (p = 0.01), IL-15 (p = 0.002), IP-10 (p = 0.019), MIG (p = 0.044) and MIP-1α (p = 0.033) maintained their significance, and IL-12 was negatively associated with positivity of *B*. *bacilliformis* by RT-PCR (p = 0.017).

**Fig 2:**
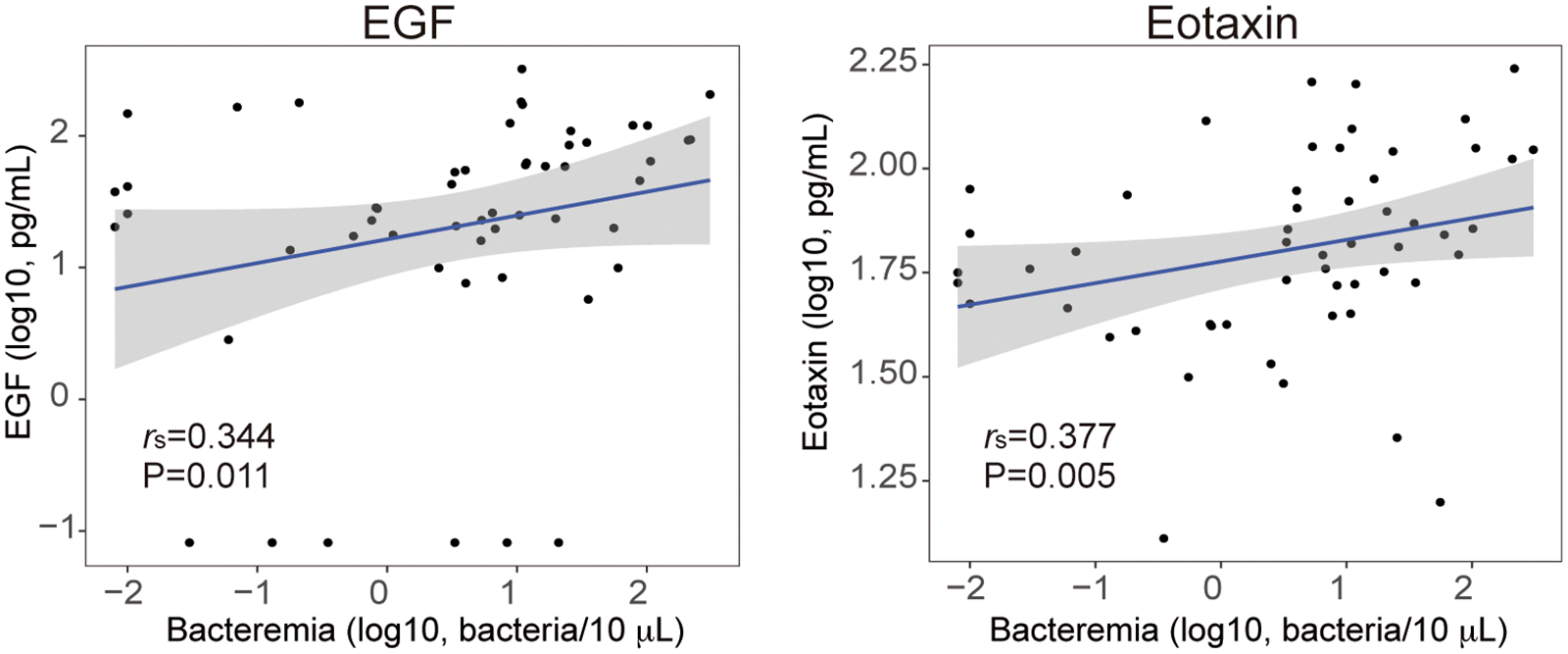
Correlations of eotaxin and EGF concentrations with *B*. *bacilliformis* bacteremia by RT-PCR. Scatter plots of subjects with detectable bacteremia by RT-PCR. Only biomarkers with statistical significant correlations with antibody levels are shown. *r*_s_ and P-values were computed through Spearman rank correlations. The grey area shows the 95% confidence interval for predictions from the linear model.

When we analyzed the impact of bacteremia on marker levels we only found bacteremia to be positively associated with EGF and eotaxin levels with moderate correlations (Fig 2). In unadjusted linear models, only eotaxin was statistically associated with bacteremia, but lost its significance after adjusting for age and area (S3 Table).

### Multi-marker associations to presence of bacteremia

**Fig 3:**
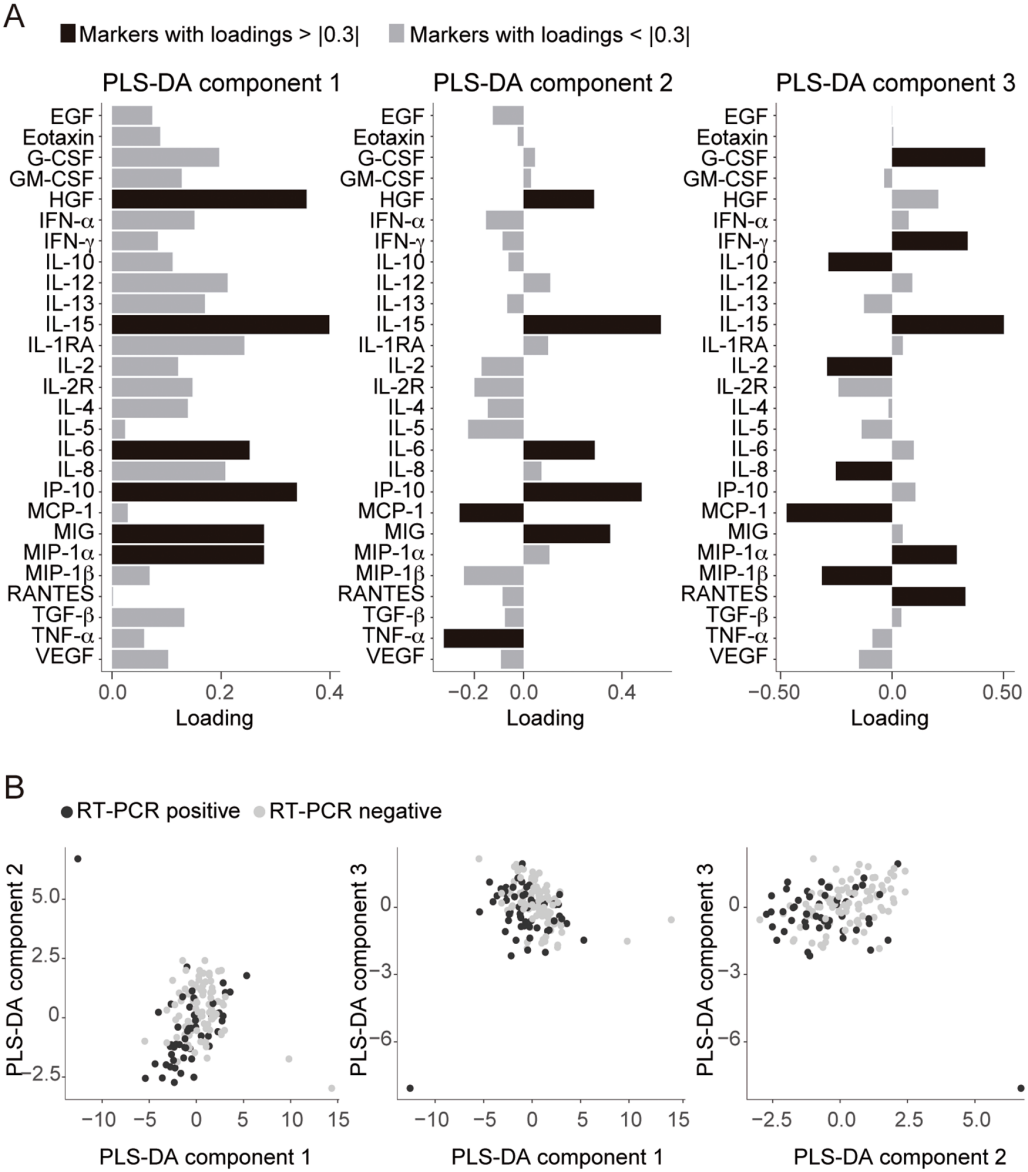
Combinations of markers associated with detectable *B*. *bacilliformis* bacteremia obtained through partial least squares discriminant analysis (PLS-DA). (A) Graphs of marker loadings to the 3 components of the PLS-DA. Bars quantify the importance (loadings) of each marker for the specific PLS-DA components that were significantly associated with RT-PCR results. Biomarkers that substantially contributed to the components (loadings > |0.3|) are highlighted in black. (B) PLS-DA plots representing each sample (dots) with respect the 3 first PLS-DA components.

We identified several components from a PLS-DA analysis that were positively and independently associated with RT-PCR results in logistic regression models. The most predictive components (components 1, 2 and 3) are shown in Fig 3A. The biomarkers that contributed more to the components were the ones with higher loadings in the different components (relevant biomarkers were considered with loadings higher than 0.3 or lower than -0.3). In component 1, the most relevant biomarkers were HGF, IL-15, IL-6, IP-10, MIG and MIP-1α, all with positive loadings; in component 2, HGF, IL-15, IL-6, IP-10 and MIG with positive loadings and MCP-1 and TNF with negative loadings, suggesting a different association with RT-PCR data. Finally, in component 3, G-CSF, INF-γ, IL-15, MIP-1α and RANTES with positive loadings, and IL-10, IL-2, IL-8 and MCP-1, MIP-1β with negative loadings (Fig 3A).

Both simple (component 1 coefficient (coef) = -0.26 p = 0.005, component 2 coef = -0.49 p = 0.001, and component 3 coef = -0.398 p = 0.029) and multivariable (component 1 coef = -0.441 p = 0.033, component 2 coef = -0.466 p = 0.007; and component 3 coef = -0.23 p = 0.010, respectively) logistic models showed a significant association between components and *B*. *bacilliformis* RT-PCR positivity. Although samples did not clearly cluster by RT-PCR results, as shown in the plots of individual samples with respect to the first 3 components (Fig 3B), the multivariable model resulting from the PLS-DA had a moderate discriminatory ability with an AUC of 0.71.

### Effect of *B*. *bacilliformis* IgM and IgG levels and seropositivity on biomarker concentrations

**Fig 4:**
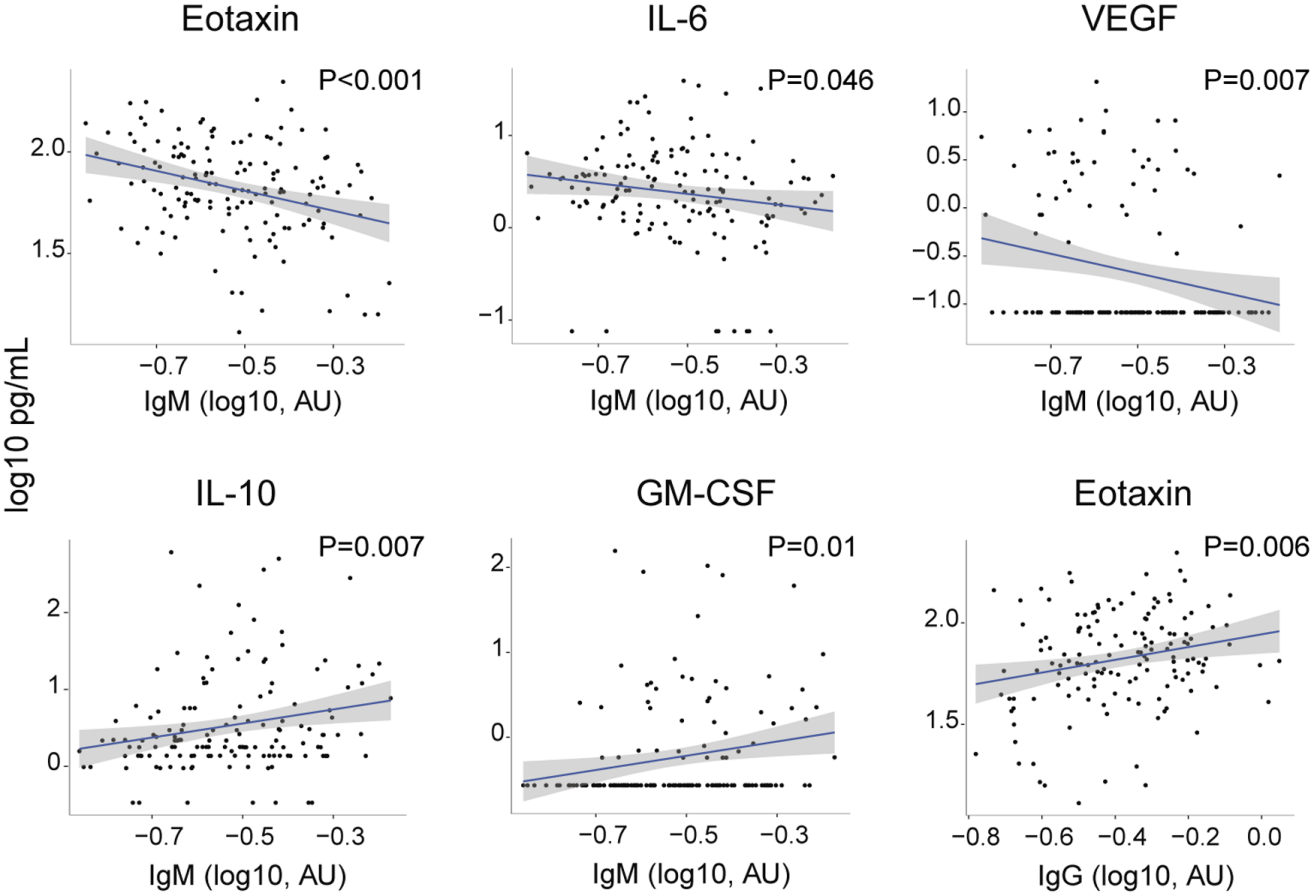
Correlations of serum biomarker concentrations and IgM and IgG levels against *B*. *bacilliformis*. Only biomarkers with statistical significant associations with antibody levels are shown. P-values were computed through unadjusted linear regressions. The grey area shows the 95% confidence interval for predictions from the linear model.

**Fig 5:**
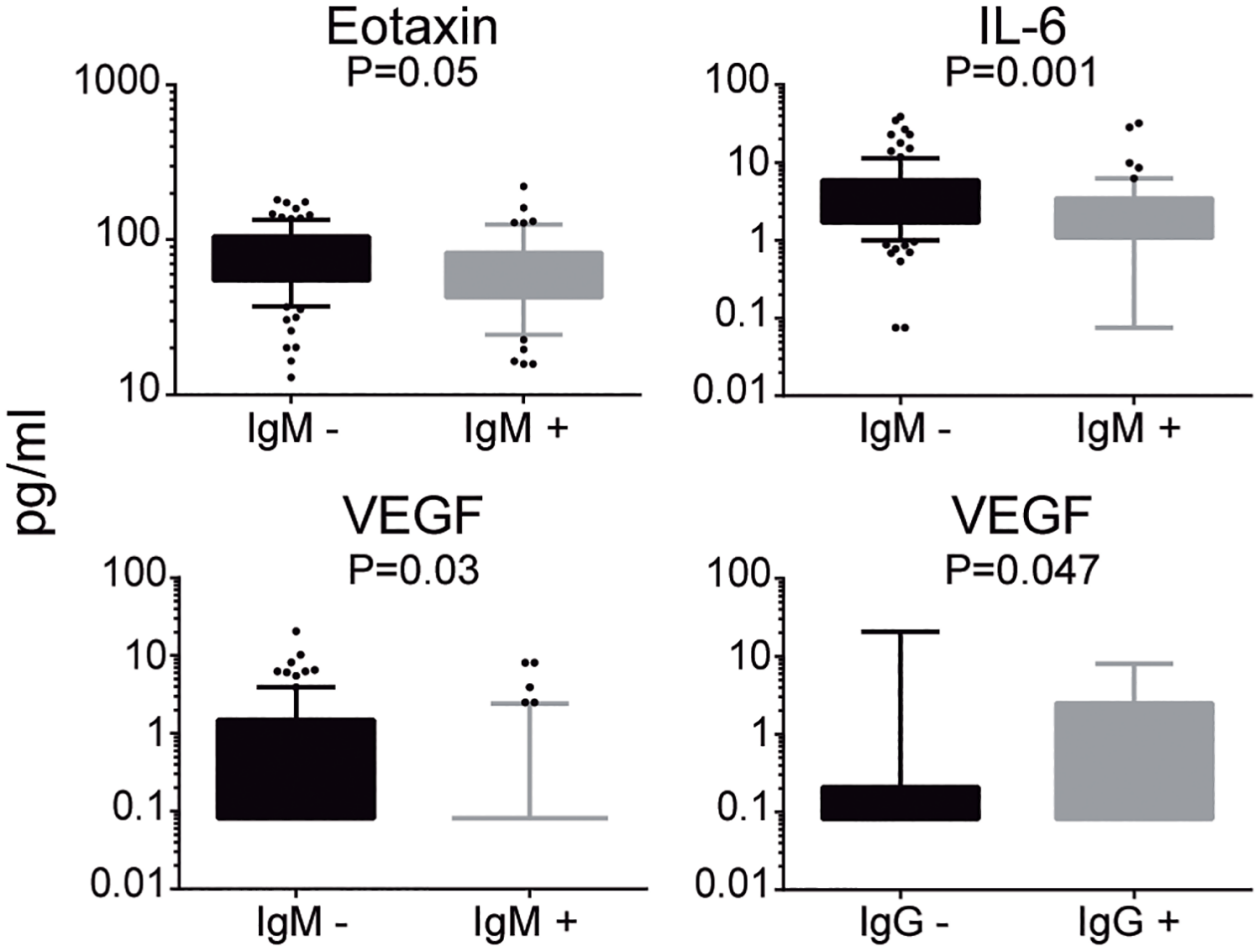
Serum biomarker concentrations according to *B*. *bacilliformis* IgM and IgG serostatus. Boxplots illustrate the 25^th^ and 75^th^ quartiles, and whiskers the percentile 10–90 of biomarkers significantly associated with IgM or IgG seropositivity. P-values were computed through unadjusted linear regressions.

We analyzed the association of IgM and IgG levels and seropositivity with biomarker levels to determine the impact of a recent acute infection and past exposure/immunity, respectively. On the one hand, IgM levels were inversely correlated with eotaxin, IL-6 and VEGF levels (p<0.0001, p = 0.046 and p = 0.007, respectively; Fig 4 and S4 Table) and consistently IgM seropositive individuals had lower levels of these three markers (p = 0.05, p = 0.001 and p = 0.03, respectively) (Fig 5 and S5 Table). On the other hand, IL-10 and GM-CSF concentrations showed to be positively associated with levels of IgM (p = 0.007 and p = 0.01, respectively; Fig 4 and S4 Table). When adjusting the analysis by age and area, the association of IL-6 with IgM levels and eotaxin with IgM seropositivity lost its significance (S4 and S5 Tables).

Regarding marker concentrations according to IgG seroprevalence, a significant difference was detected only for VEGF, which showed higher levels in the IgG seropositive group (p = 0.047, Fig 5, S6 Table). In addition, IgG levels were positively associated with levels of eotaxin (p = 0.006, Fig 4 and S7 Table). No other statistically significant associations were found. After adjusting by age and area, VEGF was positively associated with IgG levels, but eotaxin lost its significance (S7 Table).

Despite the statistical significance of these associations, correlations between biomarkers and IgM and IgG levels were moderate (Table 2).

**Table 2:** Spearman correlations between biomarker concentrations and IgM and IgG levels for biomarkers found to be associated with antibody responses.

Biomarkers	*r*_s_	P-value
IgM levels		
Eotaxin	-0.28	0.007
GM-CSF	0.26	0.002
IL-6	-0.21	0.011
IL-10	0.21	0.01
VEGF	-0.23	0.005
IgG levels		
Eotaxin	0.2	0.015

## Discussion

Host-pathogen relations established during either symptomatic or asymptomatic *B*. *bacilliformis* infections remain understudied. Nonetheless, the knowledge of the host responses to this infection and the subsequent microorganism evasion strategies are crucial to understand the pathogenesis and advance towards CD control that will precede any eradication attempt. In this context, we analyzed serum samples from both post-outbreak and endemic areas collected in 2014 to determine, for the first time, serum biomarkers associated to non-acute infection of *B*. *bacilliformis* and provide some insights into the immune response to this pathogen.

In endemic regions, an inverse correlation between age and CD incidence has been described, associated to the acquisition of partial immunological protection possibly due to antibody immunity [23]. As described in our previous work that included the samples of this study, the highest levels of IgM were found in the youngest population (under 25 years old), whereas IgG was more elevated in older population (≥25 years old) [16], reflecting increased exposure and development of immunity with age. Moreover, in traditionally endemic areas, as Huancabamba, IgG levels were higher, probably as a result of higher exposure [16]. According to previous studies, the immunological response during the acute phase is characterized by an increase in IgM levels, whereas 2 to 4 weeks later there is a significant increase of both IgG and IgM levels [24]. On the contrary, in chronic phases only a slight increase in antibody levels has been observed [24]. In our previous study, we did not find IgM and IgG levels to *B*. *bacilliformis* lysate to be associated with presence of bacteremia [16], therefore IgM would be considered a marker of recent acute-infection, whereas IgG would be a marker of past exposure and immunity. The lack of association between antibody seropositivity and levels with asymptomatic infection underscores the need to find other biomarkers such as cytokines that could identify these infections.

Lower levels of HGF, IL-12, IL-15, IL-6 (unadjusted analysis), IP-10, MIG and MIP-1α, were found in the bacteremic group. HGF is a growth factor with pleiotropic function and it is produced in response to the pro-inflammatory cytokine IL-1 in infectious diseases [25]. IL-15 induces T and NK cell proliferation and activation, and promotes the production of T_H_1 and pro-inflammatory cytokines and chemokines such as IFN-γ and MIP-1α [26]. IP-10 is also a pro-inflammatory chemokine secreted in response to IFN-γ and upregulated during acute response to infection [26]. The chemokine MIP-1α is crucial for inflammation and it is also related to synthesis of T_H_1 and pro-inflammatory cytokines [27]. We found higher levels of IL-12 with lower age, in line with previous studies in other infectious diseases, as malaria [28]. Analysis adjusting by age revealed that IL-12 was negatively associated to detection of bacteremia. IL-12 is a key T_H_1 cytokine produced mainly during innate immune response by stimulated dendritic cells, it regulates their maturation and induces IFN-γ production by T cells and NK cells as well as their activation [29]. Consistently, in multi-marker analysis we found that the most relevant markers associated with presence of bacteremia were HGF, IL-15, IL-6, IP-10, MIG and MIP-1-α and additionally, G-CSF, IFN-γ, RANTES. In the opposite direction, reflecting positive associations, we found IL-10, IL-2, IL-8, MCP-1, MIP-1β and TNF. G-CSF is a growth factor that induces the proliferation, differentiation and activation of granulocytes and it is increased in acute infections [30]. IL-2 is an important cytokine involved in many immune processes and responsible of T cell proliferation, response and induction of memory and TNF is a key T_H_1 cytokine [26]. IL-8, MIG, RANTES, MCP-1 and MIP-1β are chemokines involved in pro-inflammatory responses, although they differ on the cell types that produce them and that are targeted. Interestingly, IL-8 and MCP-1 are considered angiogenic chemokines, whereas other chemokines such as IP-10 and MIG are considered angiostatic [31]. On the other hand, IL-10 is a well-known anti-inflammatory cytokine [26]. Therefore, in non-acute infection several markers related to T_H_1 and pro-inflammatory responses were diminished, whereas IL-10 was increased in multi-marker analysis, suggesting some immunosuppression in *B*. *bacilliformis* carriers. IL-10 is upregulated in pro-inflammatory processes and limits and suppresses T cell activation and pro-inflammatory cytokines [32] to avoid an exacerbated immune response that could damage the host, but this may result in impaired pathogen control [32]. In addition, IL-10 has been associated to apoptosis of dendritic cells and immune impairment in malaria infection [33]. High levels of IL-10 have been observed in patients who presented severe sepsis, mainly by gram-negative bacteria and, importantly, IL-10 has been previously associated with *Bartonella* infections. It is hypothesized that IL-10 induced immunosuppression is key in the development of severe Bartonellosis [34]. However, anti-inflammatory effects of IL-10 have also been described in mild *Bartonella* infections where this cytokine would limit immunopathogenesis at the expense of allowing persistent infections [35]. Probably, as occurs in *B*. *quintana* infection [36], there is an IL-10 increased production that is associated with an attenuated inflammatory profile in infected compared to non-infected individuals.

IgM levels correlated with low levels of some pro-inflammatory markers and higher IL-10 levels, probably still reflecting an immunosuppression of the acute phase of the infection. Indeed, an impairment of the cellular immune response in acute infection has been previously described [37]. These results, together with the associations with bacteremia described above, support the hypothesis that *B*. *bacilliformis* induces a systemic immunosuppression that could last a long time after the acute infection and could be maintained during the chronic phase.

GM-CSF was also positively associated with IgM levels. GM-CSF is a growth factor that stimulates production and antibacterial functions of neutrophils, monocytes and macrophages [26]. It has an important role in acute infections [38] and has been found elevated in sepsis [39]. The higher levels found in correlation with IgM probably reflects levels still elevated from the acute infection. It seems that concurrent pro-inflammatory and anti-inflammatory responses in acute Bartonellosis may be similar to what occurs in other sepsis [40].

Interestingly, we found an association between increased bacteremia and increased levels of EGF and eotaxin, although we did not find any differences in the concentration of these markers in infected and non-infected subjects. EGF is a growth factor associated with endothelial cells and related to angiogenic processes [41]. It could be involved in the chronic phase of *B*. *bacilliformis* infection, when the infection promotes cutaneous angiogenesis that involves proliferation of endothelial cells, resulting in a series of cutaneous lesions [42]. These cutaneous lesions are characterized by small vessels coated with endothelial cells and are a junction of polymorphonuclear neutrophils, macrophages, endothelial cells and bacteria [42]. Eotaxin is a selective chemoattractant for eosinophils, but it is also involved in recruitment and activation of other immune cells in inflamed tissue [26]. Importantly, this chemokine is produced by macrophages, fibroblasts and endothelial cells [43] and also mediates angiogenesis [44]. Therefore it may also be a relevant marker in the angiogenic processes in Bartonellosis. To our knowledge, eotaxin has not been studied before in any *Bartonella* infection, while the induction of VEGF by *Bartonella*, another potent angiogenic factor secreted by endothelial cells and macrophages from the lesions [45–46] has been described. The presence of *B*. *henselae*, specifically the pili, was associated with host cell VEGF production [46] and VEGF-stimulated endothelial cells promoted the growth of *B*. *henselae* through the activation of hypoxia-inducible factor-1 [47–48]. Simultaneously, *B*. *henselae* also inhibited cell apoptosis, inducing endothelial proliferation [49] and allowing *Bartonella* proliferation in these cells. Despite none of the analyzed samples belongs to patients with Peruvian warts, this is with cutaneous lesions, we found that VEGF levels were higher in IgG seropositive individuals, and were inversely associated with IgM levels. Similarly, eotaxin was positively associated with IgG levels, but inversely associated with IgM levels and seropositivity. This, together with its correlation with bacteremia, suggest that this chemokine may be suppressed only in acute infection and induced by bacteremia in tissues during the chronic phase. Importantly, we also found the angiogenic chemokines IL-8 and MCP-1 and IP-10, MIP-1α and RANTES, which are induced by *B*. *henselae* in endothelial cells [47], macrophages [50] and in infected human myeloid angiogenic cells that differentiates to tumour associated macrophages [51], to be associated with presence of bacteremia in multivariate analysis. Thus, we hypothesize that EGF, eotaxin, VEGF and other angiogenic chemokines described in previous in vitro studies with *B*. *henselae* [46–48,50–51] could be markers of chronic *B*. *bacilliformis* infection, and they could predict the appearance of cutaneous lesions. IgG levels in semi-immune individuals could be forcing *Bartonella* to hide into the endothelium and epidermis, where it would induce vasculogenesis and angiogenesis through the production of eotaxin, EGF, VEGF and other angiogenic chemokines that would ultimately result in the Peruvian warts [45–52]. *Bartonella* hiding, in conjunction with the systemic immunosupression that would avoid high antibody responses, could explain the observed lack of association between IgG response and RT-PCR results in this study.

A limitation of our study was the lack of samples of acutely infected subjects or chronically infected individuals with Peruvian warts, to clarify the cytokine profile in each stage of the infection. In addition, the cross-sectional design of the study did not allow addressing kinetics of biomarkers and immune responses and associations with disease progression.

In accordance with our results, we speculate that *B*. *bacilliformis* induces an immunosuppression caused at least in part by elevated levels of IL-10 in the acute phase, and this is maintained in later phases with low levels of bacteremia, as occurs in *B*. *quintana*. Due to this transient immune paralysis, levels of some T_H_1-related and pro-inflammatory cytokines are reduced, helping the establishment and persistence of the infection at low levels of bacteremia. This attenuated response in bacteremic individuals could be the result of mechanisms of immune tolerance defined as the absence of a specific immune response to an antigen. *B*. *bacilliformis* could be triggering these mechanisms as a strategy of immune evasion, and in this manner allowing the subsequent transmission. In addition, our findings support the idea that immune pressure mediated by IgG responses could be forcing *B*. *bacilliformis* to hide and replicate in endothelial cells [49], slowly growing inside cells, isolated from the host immune response, enabling bacteria survival. Accordingly, angiogenic growth factors and chemokines positively associated with bacteremia and IgG levels could be useful as biomarkers of asymptomatic and chronic infection. Despite the moderate discriminatory ability of the markers identified and the considerable likelihood of false discovery rate, future studies designed and powered to evaluate biomarkers of *B*. *bacilliformis* infection at different stages should address these markers and novel markers in combination.

## Supporting information

S1 Dataset Cytokine, chemokine and growth factor concentrations and other study data for each subject. (XLSX)

S1 Table Effect of age and area on marker levels. (DOCX)

S2 Table Unadjusted and adjusted analysis of the effect of RT-PCR on marker levels. (DOCX)

S3 Table Unadjusted and adjusted analysis of the effect of bacteremia on marker levels. (DOCX)

S4 Table Unadjusted and adjusted analysis of the effect of IgM levels on marker levels. (DOCX)

S5 Table Unadjusted and adjusted analysis of the effect of IgM seropositivity on marker levels. (DOCX)

S6 Table Unadjusted and adjusted analysis of the effect of IgG seropositivity on marker levels. (DOCX)

S7 Table Unadjusted and adjusted analysis of the effect of IgG levels on marker levels. (DOCX)
