# Supplementary material for: Immunosuppressive and angiogenic cytokine profile associated with Bartonella bacilliformis infection in post-outbreak and endemic areas of Carrion's disease in Peru
Source: PLoS Negl Trop Dis. 2017 Jun 19;11(6):e0005684. doi: 10.1371/journal.pntd.0005684 (PMC5491314; doi:10.1371/journal.pntd.0005684)
Supplement: S1 Table — (DOCX) [file pntd.0005684.s002.docx]

**S1 Table.** Effect of age and area on marker levels.

|  | **Effect of age** | | | **Effect of post-outbreak areas compared to endemic area** | | |
| --- | --- | --- | --- | --- | --- | --- |
|  | **Coefficient** | **95% CI** | **p-value** ^a^ | **Coefficient** | **95% CI** | **p-value** ^b^ |
| **EGF** | -0.133 | -0.591; 0.326 | 0.568 | -0.663 | -1.067; -0.259 | 0.001 |
| **eotaxin** | 0.293 | 0.181; 0.405 | <0.001 | -0.103 | -0.213; 0.007 | 0.066 |
| **G-CSF** | -0.087 | -0.207; 0.033 | 0.154 | -0.065 | -0.175; 0.045 | 0.244 |
| **GM-CSF** | -0.155 | -0.467; 0.157 | 0.329 | 0.205 | -0.079; 0.489 | 0.155 |
| **HGF** | 0.077 | -0.019; 0.174 | 0.116 | -0.028 | -0.117; 0.061 | 0.534 |
| **IFN-α** | -0.029 | -0.101; 0.043 | 0.429 | -0.012 | -0.078; 0.054 | 0.73 |
| **IFN-γ** | -0.032 | -0.134; 0.069 | 0.528 | -0.033 | -0.125; 0.06 | 0.485 |
| **IL-10** | -0.259 | -0.583; 0.064 | 0.116 | 0.244 | -0.052; 0.539 | 0.105 |
| **IL-12** | -0.085 | -0.127; -0.043 | <0.001 | -0.002 | -0.042; 0.039 | 0.925 |
| **IL-13** | 0.038 | -0.137; 0.213 | 0.67 | 0.017 | -0.143; 0.177 | 0.835 |
| **IL-15** | 0.023 | -0.463; 0.508 | 0.927 | 0.265 | -0.177; 0.706 | 0.238 |
| **IL-1RA** | 0.03 | -0.161; 0.22 | 0.759 | 0.034 | -0.14; 0.208 | 0.699 |
| **IL-2** | -0.182 | -0.342; -0.022 | 0.026 | 0.061 | -0.087; 0.209 | 0.417 |
| **IL-2R** | -0.041 | -0.122; 0.039 | 0.311 | 0.011 | -0.062; 0.085 | 0.763 |
| **IL-4** | -0.064 | -0.196; 0.067 | 0.335 | -0.066 | -0.194; 0.062 | 0.31 |
| **IL-5** | -0.23 | -0.527; 0.066 | 0.127 | 0.005 | -0.268; 0.278 | 0.97 |
| **IL-6** | 0.362 | 0.095; 0.629 | 0.008 | 0.165 | -0.084; 0.413 | 0.192 |
| **IL-8** | 0.073 | -0.075; 0.22 | 0.333 | -0.013 | -0.148; 0.122 | 0.846 |
| **IP-10** | 0.038 | -0.088; 0.164 | 0.552 | 0.137 | 0.022; 0.252 | 0.019 |
| **MCP-1** | 0.167 | 0.062; 0.272 | 0.002 | 0.013 | -0.086; 0.113 | 0.79 |
| **MIG** | 0.274 | -0.124; 0.672 | 0.176 | -0.117 | -0.482; 0.248 | 0.527 |
| **MIP-1α** | 0.007 | -0.055; 0.069 | 0.813 | 0.001 | -0.056; 0.057 | 0.979 |
| **MIP-1β** | 0.027 | -0.085; 0.139 | 0.637 | 0.001 | -0.102; 0.104 | 0.979 |
| **RANTES** | 0.06 | -0.073; 0.194 | 0.372 | -0.027 | -0.149; 0.094 | 0.657 |
| **TNF** | -0.03 | -0.146; 0.085 | 0.604 | -0.114 | -0.218; -0.01 | 0.032 |
| **VEGF** | 0.163 | -0.197; 0.524 | 0.372 | 0.071 | -0.259; 0.401 | 0.671 |

Abbreviations: CI, confidence interval

**^a^** P-values were computed through linear regressions using log10-transformed marker concentration as outcome and log10-transformed age as the predictor variable.

^b^ P-values were computed through linear regressions using log10-transformed marker concentration as outcome and area as the predictor variable.
