# Supplementary material for: Immunosuppressive and angiogenic cytokine profile associated with Bartonella bacilliformis infection in post-outbreak and endemic areas of Carrion's disease in Peru
Source: PLoS Negl Trop Dis. 2017 Jun 19;11(6):e0005684. doi: 10.1371/journal.pntd.0005684 (PMC5491314; doi:10.1371/journal.pntd.0005684)
Supplement: S2 Table — (DOCX) [file pntd.0005684.s003.docx]

**S2 Table.** Unadjusted and adjusted analysis of the effect of RT-PCR on marker levels.

|  | **Unadjusted model** | | | | **Models adjusted by age and area** | | | |
| --- | --- | --- | --- | --- | --- | --- | --- | --- |
|  | **Coefficient** | **95% CI** | **p-value** ^a^ | **BH ^b^** | **Coefficient** | **95% CI** | **p-value** ^a^ | **BH ^b^** |
| **EGF** | -0.088 | -0.393; 0.217 | 0.569 | 0.705 | -0.136 | -0.438; 0.165 | 0.374 | 0.54 |
| **eotaxin** | -0.028 | -0.109; 0.053 | 0.496 | 0.671 | -0.004 | -0.079; 0.071 | 0.913 | 0.913 |
| **G-CSF** | -0.062 | -0.141; 0.018 | 0.128 | 0.333 | -0.076 | -0.157; 0.005 | 0.065 | 0.213 |
| **GM-CSF** | -0.104 | -0.312; 0.104 | 0.324 | 0.562 | -0.117 | -0.328; 0.094 | 0.274 | 0.433 |
| **HGF** | -0.091 | -0.154; -0.027 | **0.005** | 0.068 | -0.086 | -0.15; -0.021 | **0.01** | 0.12 |
| **IFN-α** | -0.028 | -0.076; 0.019 | 0.243 | 0.508 | -0.033 | -0.082; 0.016 | 0.188 | 0.432 |
| **IFN-γ** | -0.022 | -0.089; 0.045 | 0.516 | 0.671 | -0.028 | -0.097; 0.041 | 0.417 | 0.57 |
| **IL-10** | -0.094 | -0.311; 0.122 | 0.391 | 0.598 | -0.119 | -0.336; 0.099 | 0.283 | 0.433 |
| **IL-12** | -0.024 | -0.054; 0.005 | 0.1 | 0.31 | -0.034 | -0.063; -0.006 | **0.017** | 0.12 |
| **IL-13** | -0.078 | -0.194; 0.038 | 0.187 | 0.443 | -0.072 | -0.191; 0.047 | 0.236 | 0.433 |
| **IL-15** | -0.504 | -0.816; -0.192 | **0.002** | 0.045 | -0.499 | -0.818; -0.179 | **0.002** | 0.064 |
| **IL-1RA** | -0.12 | -0.246; 0.005 | 0.059 | 0.221 | -0.121 | -0.25; 0.008 | 0.065 | 0.213 |
| **IL-2** | -0.051 | -0.159; 0.057 | 0.349 | 0.568 | -0.068 | -0.176; 0.04 | 0.216 | 0.432 |
| **IL-2R** | -0.031 | -0.084; 0.022 | 0.254 | 0.508 | -0.035 | -0.09; 0.019 | 0.202 | 0.432 |
| **IL-4** | -0.051 | -0.144; 0.042 | 0.282 | 0.524 | -0.05 | -0.14; 0.039 | 0.267 | 0.433 |
| **IL-5** | -0.018 | -0.217; 0.18 | 0.855 | 0.889 | -0.039 | -0.242; 0.163 | 0.7 | 0.793 |
| **IL-6** | -0.18 | -0.36; 0.000 | **0.05** | 0.216 | -0.145 | -0.324; 0.035 | 0.115 | 0.331 |
| **IL-8** | -0.08 | -0.178; 0.018 | 0.107 | 0.31 | -0.075 | -0.175; 0.025 | 0.143 | 0.371 |
| **IP-10** | -0.113 | -0.196; -0.03 | **0.008** | 0.07 | -0.1 | -0.183; -0.017 | **0.019** | 0.12 |
| **MCP-1** | -0.008 | -0.081; 0.064 | 0.825 | 0.889 | 0.011 | -0.061; 0.083 | 0.756 | 0.819 |
| **MIG** | -0.291 | -0.553; -0.029 | **0.03** | 0.156 | -0.275 | -0.543; -0.008 | **0.044** | 0.189 |
| **MIP-1α** | -0.045 | -0.085; -0.004 | **0.03** | 0.156 | -0.045 | -0.087; -0.004 | **0.033** | 0.172 |
| **MIP-1β** | -0.02 | -0.095; 0.055 | 0.596 | 0.705 | -0.015 | -0.092; 0.062 | 0.702 | 0.793 |
| **RANTES** | -0.001 | -0.089; 0.088 | 0.99 | 0.99 | 0.006 | -0.085; 0.097 | 0.901 | 0.913 |
| **TNF** | -0.018 | -0.095; 0.059 | 0.651 | 0.736 | -0.028 | -0.106; 0.05 | 0.482 | 0.619 |
| **VEGF** | -0.097 | -0.337; 0.143 | 0.428 | 0.618 | -0.084 | -0.33; 0.162 | 0.5 | 0.619 |

Abbreviations: CI, confidence interval

**^a^** P-values were computed through linear regressions using log10-transformed marker concentration as outcome and RT-PCR results as the predictor variable.

^b^ P-values were adjusted by multiple testing using a Benjamini-Hochberg approach.
