# Supplementary material for: Immunosuppressive and angiogenic cytokine profile associated with Bartonella bacilliformis infection in post-outbreak and endemic areas of Carrion's disease in Peru
Source: PLoS Negl Trop Dis. 2017 Jun 19;11(6):e0005684. doi: 10.1371/journal.pntd.0005684 (PMC5491314; doi:10.1371/journal.pntd.0005684)
Supplement: S3 Table — (DOCX) [file pntd.0005684.s004.docx]

**S3 Table**. Unadjusted and adjusted analysis of the effect of bacteremia on marker levels.

|  | **Unadjusted model** | | | | **Models adjusted by age and area** | | | |
| --- | --- | --- | --- | --- | --- | --- | --- | --- |
|  | **Coefficient** | **95% CI** | **p-value** ^a^ | **BH ^b^** | **Coefficient** | **95% CI** | **p-value** ^a^ | **BH ^b^** |
| **EGF** | 0.18 | -0.029; 0.39 | 0.09 | 0.719 | 0.165 | -0.045; 0.376 | 0.122 | 0.737 |
| **eotaxin** | 0.052 | 0.001; 0.103 | **0.044** | 0.719 | 0.045 | -0.006; 0.096 | 0.082 | 0.737 |
| **G-CSF** | -0.007 | -0.082; 0.069 | 0.86 | 0.991 | -0.012 | -0.088; 0.064 | 0.749 | 0.994 |
| **GM-CSF** | 0.046 | -0.069; 0.161 | 0.429 | 0.991 | 0.069 | -0.041; 0.179 | 0.214 | 0.796 |
| **HGF** | 0.023 | -0.02; 0.065 | 0.293 | 0.991 | 0.017 | -0.026; 0.061 | 0.427 | 0.994 |
| **IFN-α** | -0.004 | -0.04; 0.033 | 0.841 | 0.991 | -0.004 | -0.042; 0.033 | 0.82 | 0.994 |
| **IFN-γ** | 0.001 | -0.069; 0.071 | 0.974 | 0.991 | 0.002 | -0.07; 0.075 | 0.951 | 0.994 |
| **IL-10** | 0.033 | -0.101; 0.167 | 0.626 | 0.991 | 0.064 | -0.067; 0.195 | 0.329 | 0.994 |
| **IL-12** | -0.001 | -0.019; 0.018 | 0.933 | 0.991 | 0.001 | -0.017; 0.019 | 0.893 | 0.994 |
| **IL-13** | 0.005 | -0.077; 0.086 | 0.908 | 0.991 | -0.002 | -0.086; 0.082 | 0.956 | 0.994 |
| **IL-15** | 0.146 | -0.062; 0.354 | 0.166 | 0.719 | 0.159 | -0.058; 0.376 | 0.147 | 0.737 |
| **IL-1RA** | 0.059 | -0.024; 0.142 | 0.159 | 0.719 | 0.06 | -0.027; 0.147 | 0.17 | 0.737 |
| **IL-2** | 0.053 | -0.02; 0.126 | 0.15 | 0.719 | 0.061 | -0.013; 0.135 | 0.102 | 0.737 |
| **IL-2R** | 0.007 | -0.026; 0.04 | 0.675 | 0.991 | 0.01 | -0.024; 0.044 | 0.558 | 0.994 |
| **IL-4** | 0.003 | -0.044; 0.049 | 0.905 | 0.991 | -0.002 | -0.047; 0.042 | 0.915 | 0.994 |
| **IL-5** | -0.001 | -0.125; 0.124 | 0.991 | 0.991 | 0.018 | -0.107; 0.143 | 0.773 | 0.994 |
| **IL-6** | 0.006 | -0.127; 0.139 | 0.929 | 0.991 | 0.015 | -0.123; 0.153 | 0.827 | 0.994 |
| **IL-8** | 0.002 | -0.073; 0.076 | 0.968 | 0.991 | -0.002 | -0.08; 0.076 | 0.954 | 0.994 |
| **IP-10** | 0.019 | -0.035; 0.073 | 0.488 | 0.991 | 0.024 | -0.033; 0.08 | 0.404 | 0.994 |
| **MCP-1** | -0.01 | -0.056; 0.036 | 0.664 | 0.991 | -0.013 | -0.06; 0.035 | 0.595 | 0.994 |
| **MIG** | 0.074 | -0.174; 0.322 | 0.551 | 0.991 | 0.048 | -0.208; 0.305 | 0.706 | 0.994 |
| **MIP-1α** | 0 | -0.02; 0.02 | 0.973 | 0.991 | 0 | -0.021; 0.021 | 0.999 | 0.999 |
| **MIP-1β** | 0.005 | -0.043; 0.052 | 0.846 | 0.991 | 0.005 | -0.044; 0.054 | 0.847 | 0.994 |
| **RANTES** | 0.011 | -0.055; 0.077 | 0.741 | 0.991 | 0.016 | -0.052; 0.084 | 0.647 | 0.994 |
| **TNF** | 0.013 | -0.038; 0.064 | 0.605 | 0.991 | 0.01 | -0.042; 0.062 | 0.708 | 0.994 |
| **VEGF** | 0.119 | -0.023; 0.261 | 0.097 | 0.719 | 0.141 | -0.003; 0.285 | 0.055 | 0.737 |

Abbreviations: CI, confidence interval

^a^ P-values were computed through linear regressions using log10-transformed marker concentration as outcome and log10-transformed bacteremia as the predictor variable.

**^b^** P-values were adjusted by multiple testing using a Benjamini-Hochberg approach.
