# Supplementary material for: Immunosuppressive and angiogenic cytokine profile associated with Bartonella bacilliformis infection in post-outbreak and endemic areas of Carrion's disease in Peru
Source: PLoS Negl Trop Dis. 2017 Jun 19;11(6):e0005684. doi: 10.1371/journal.pntd.0005684 (PMC5491314; doi:10.1371/journal.pntd.0005684)
Supplement: S4 Table — (DOCX) [file pntd.0005684.s005.docx]

**S4 Table**. Unadjusted and adjusted analysis of the effect of IgM levels on marker levels.

|  | **Unadjusted model** | | | | **Models adjusted by age and area** | | | |
| --- | --- | --- | --- | --- | --- | --- | --- | --- |
|  | **Coefficient** | **95% CI** | **p-value** ^a^ | **BH ^b^** | **Coefficient** | **95% CI** | **p-value** ^a^ | **BH ^b^** |
| **EGF** | -0.105 | -1.062; 0.852 | 0.829 | 0.928 | -0.159 | -1.169; 0.85 | 0.755 | 0.988 |
| **eotaxin** | -0.491 | -0.732; -0.249 | **<0.001** | 0.002 | -0.29 | -0.535; -0.044 | **0.021** | 0.202 |
| **G-CSF** | 0.067 | -0.185; 0.319 | 0.598 | 0.856 | 0.006 | -0.267; 0.279 | 0.966 | 0.988 |
| **GM-CSF** | 0.84 | 0.201; 1.479 | **0.01** | 0.067 | 0.805 | 0.111; 1.499 | **0.023** | 0.202 |
| **HGF** | -0.15 | -0.352; 0.052 | 0.145 | 0.419 | -0.095 | -0.315; 0.124 | 0.392 | 0.928 |
| **IFN-α** | 0.036 | -0.115; 0.187 | 0.641 | 0.856 | 0.017 | -0.148; 0.182 | 0.843 | 0.988 |
| **IFN-γ** | -0.01 | -0.221; 0.201 | 0.927 | 0.931 | -0.039 | -0.27; 0.191 | 0.738 | 0.988 |
| **IL-10** | 0.914 | 0.249; 1.579 | **0.007** | 0.064 | 0.782 | 0.065; 1.5 | **0.033** | 0.213 |
| **IL-12** | 0.059 | -0.033; 0.151 | 0.21 | 0.546 | -0.011 | -0.107; 0.085 | 0.825 | 0.988 |
| **IL-13** | 0.168 | -0.198; 0.533 | 0.367 | 0.592 | 0.241 | -0.157; 0.639 | 0.233 | 0.864 |
| **IL-15** | 0.444 | -0.567; 1.456 | 0.387 | 0.592 | 0.531 | -0.569; 1.631 | 0.342 | 0.928 |
| **IL-1RA** | -0.053 | -0.45; 0.345 | 0.794 | 0.928 | -0.041 | -0.476; 0.394 | 0.852 | 0.988 |
| **IL-2** | 0.318 | -0.018; 0.653 | 0.063 | 0.273 | 0.197 | -0.166; 0.559 | 0.285 | 0.926 |
| **IL-2R** | 0.038 | -0.131; 0.206 | 0.658 | 0.856 | 0.005 | -0.179; 0.189 | 0.956 | 0.988 |
| **IL-4** | 0.053 | -0.241; 0.346 | 0.722 | 0.894 | 0.044 | -0.256; 0.343 | 0.773 | 0.988 |
| **IL-5** | 0.362 | -0.259; 0.983 | 0.251 | 0.555 | 0.217 | -0.459; 0.892 | 0.527 | 0.988 |
| **IL-6** | -0.575 | -1.139; -0.011 | **0.046** | 0.237 | -0.367 | -0.971; 0.236 | 0.231 | 0.864 |
| **IL-8** | -0.171 | -0.478; 0.137 | 0.275 | 0.555 | -0.127 | -0.463; 0.209 | 0.457 | 0.988 |
| **IP-10** | -0.222 | -0.487; 0.043 | 0.1 | 0.371 | -0.225 | -0.505; 0.056 | 0.115 | 0.599 |
| **MCP-1** | -0.174 | -0.4; 0.051 | 0.129 | 0.418 | -0.045 | -0.285; 0.195 | 0.712 | 0.988 |
| **MIG** | -0.445 | -1.278; 0.387 | 0.292 | 0.555 | -0.243 | -1.149; 0.663 | 0.597 | 0.988 |
| **MIP-1α** | -0.006 | -0.135; 0.124 | 0.931 | 0.931 | 0.001 | -0.14; 0.143 | 0.988 | 0.988 |
| **MIP-1β** | -0.124 | -0.358; 0.111 | 0.299 | 0.555 | -0.112 | -0.367; 0.144 | 0.389 | 0.928 |
| **RANTES** | -0.13 | -0.407; 0.148 | 0.358 | 0.592 | -0.088 | -0.392; 0.215 | 0.566 | 0.988 |
| **TNF** | 0.022 | -0.22; 0.264 | 0.856 | 0.928 | 0.01 | -0.25; 0.271 | 0.939 | 0.988 |
| **VEGF** | -1.011 | -1.747; -0.275 | **0.007** | 0.064 | -1.055 | -1.858; -0.251 | **0.01** | 0.202 |

Abbreviations: CI, confidence interval

^a^ P-values were computed through linear regressions using log10-transformed marker concentration as outcome and log10-transformed IgM levels as the predictor variable.

**^b^** P-values were adjusted by multiple testing using a Benjamini-Hochberg approach.
