# Supplementary material for: Immunosuppressive and angiogenic cytokine profile associated with Bartonella bacilliformis infection in post-outbreak and endemic areas of Carrion's disease in Peru
Source: PLoS Negl Trop Dis. 2017 Jun 19;11(6):e0005684. doi: 10.1371/journal.pntd.0005684 (PMC5491314; doi:10.1371/journal.pntd.0005684)
Supplement: S5 Table — (DOCX) [file pntd.0005684.s006.docx]

**S5 Table**. Unadjusted and adjusted analysis of the effect of IgM seropositivity on marker levels.

|  | **Unadjusted model** | | | | **Models adjusted by age and area** | | | |
| --- | --- | --- | --- | --- | --- | --- | --- | --- |
|  | **Coefficient** | **95% CI** | **p-value** ^a^ | **BH ^b^** | **Coefficient** | **95% CI** | **p-value** ^a^ | **BH ^b^** |
| **EGF** | 0.042 | -0.265; 0.35 | 0.785 | 0.852 | 0.009 | -0.304; 0.321 | 0.957 | 0.957 |
| **eotaxin** | -0.08 | -0.161; 0 | **0.05** | 0.402 | -0.033 | -0.11; 0.044 | 0.404 | 0.948 |
| **G-CSF** | 0.021 | -0.06; 0.102 | 0.604 | 0.852 | 0.005 | -0.079; 0.089 | 0.908 | 0.948 |
| **GM-CSF** | 0.198 | -0.01; 0.405 | 0.062 | 0.402 | 0.185 | -0.031; 0.401 | 0.093 | 0.808 |
| **HGF** | -0.036 | -0.101; 0.029 | 0.274 | 0.604 | -0.023 | -0.091; 0.045 | 0.505 | 0.948 |
| **IFN-α** | 0.009 | -0.039; 0.058 | 0.707 | 0.852 | 0.004 | -0.047; 0.055 | 0.866 | 0.948 |
| **IFN-γ** | 0.001 | -0.067; 0.069 | 0.979 | 0.979 | -0.007 | -0.078; 0.065 | 0.857 | 0.948 |
| **IL-10** | 0.187 | -0.03; 0.404 | 0.091 | 0.473 | 0.151 | -0.073; 0.375 | 0.186 | 0.948 |
| **IL-12** | 0.023 | -0.006; 0.053 | 0.122 | 0.496 | 0.008 | -0.021; 0.038 | 0.575 | 0.948 |
| **IL-13** | 0.037 | -0.081; 0.155 | 0.534 | 0.852 | 0.052 | -0.071; 0.176 | 0.402 | 0.948 |
| **IL-15** | -0.051 | -0.377; 0.275 | 0.756 | 0.852 | -0.038 | -0.379; 0.303 | 0.828 | 0.948 |
| **IL-1RA** | -0.044 | -0.171; 0.084 | 0.501 | 0.852 | -0.042 | -0.176; 0.092 | 0.539 | 0.948 |
| **IL-2** | 0.06 | -0.049; 0.168 | 0.279 | 0.604 | 0.031 | -0.081; 0.144 | 0.582 | 0.948 |
| **IL-2R** | 0.01 | -0.044; 0.064 | 0.712 | 0.852 | 0.004 | -0.053; 0.06 | 0.902 | 0.948 |
| **IL-4** | 0.013 | -0.081; 0.108 | 0.781 | 0.852 | 0.013 | -0.079; 0.106 | 0.775 | 0.948 |
| **IL-5** | 0.027 | -0.173; 0.228 | 0.787 | 0.852 | -0.012 | -0.221; 0.197 | 0.912 | 0.948 |
| **IL-6** | -0.3 | -0.477; -0.124 | **0.001** | 0.026 | -0.256 | -0.439; -0.074 | **0.006** | 0.161 |
| **IL-8** | -0.058 | -0.157; 0.04 | 0.245 | 0.604 | -0.048 | -0.152; 0.056 | 0.362 | 0.948 |
| **IP-10** | -0.065 | -0.15; 0.02 | 0.134 | 0.496 | -0.054 | -0.141; 0.033 | 0.223 | 0.948 |
| **MCP-1** | -0.045 | -0.117; 0.028 | 0.225 | 0.604 | -0.014 | -0.088; 0.06 | 0.712 | 0.948 |
| **MIG** | -0.176 | -0.443; 0.091 | 0.195 | 0.604 | -0.137 | -0.416; 0.143 | 0.335 | 0.948 |
| **MIP-1α** | 0.002 | -0.04; 0.043 | 0.939 | 0.977 | 0.004 | -0.04; 0.047 | 0.873 | 0.948 |
| **MIP-1β** | -0.038 | -0.113; 0.037 | 0.321 | 0.641 | -0.033 | -0.112; 0.046 | 0.414 | 0.948 |
| **RANTES** | -0.025 | -0.115; 0.064 | 0.58 | 0.852 | -0.015 | -0.108; 0.079 | 0.759 | 0.948 |
| **TNF** | 0.029 | -0.049; 0.106 | 0.464 | 0.852 | 0.022 | -0.058; 0.103 | 0.583 | 0.948 |
| **VEGF** | -0.264 | -0.503; -0.026 | **0.03** | 0.393 | -0.258 | -0.509; -0.008 | **0.044** | 0.566 |

Abbreviations: CI, confidence interval

^a^ P-values were computed through linear regressions using log10-transformed marker concentration as outcome and IgM seropositivity as the predictor variable.

**^b^** P-values were adjusted by multiple testing using a Benjamini-Hochberg approach.
