# Supplementary material for: Immunosuppressive and angiogenic cytokine profile associated with Bartonella bacilliformis infection in post-outbreak and endemic areas of Carrion's disease in Peru
Source: PLoS Negl Trop Dis. 2017 Jun 19;11(6):e0005684. doi: 10.1371/journal.pntd.0005684 (PMC5491314; doi:10.1371/journal.pntd.0005684)
Supplement: S6 Table — (DOCX) [file pntd.0005684.s007.docx]

**S6 Table**. Unadjusted and adjusted analysis of the effect of IgG seropositivity on marker levels.

|  | **Unadjusted model** | | | | **Models adjusted by age and area** | | | |
| --- | --- | --- | --- | --- | --- | --- | --- | --- |
|  | **Coefficient** | **95% CI** | **p-value** ^a^ | **BH ^b^** | **Coefficient** | **95% CI** | **p-value** ^a^ | **BH ^b^** |
| **EGF** | -0.026 | -0.348; 0.297 | 0.874 | 0.895 | -0.236 | -0.577; 0.105 | 0.173 | 0.557 |
| **eotaxin** | 0.069 | -0.016; 0.154 | 0.11 | 0.764 | 0.007 | -0.078; 0.092 | 0.865 | 0.973 |
| **G-CSF** | -0.031 | -0.116; 0.054 | 0.476 | 0.764 | -0.046 | -0.138; 0.047 | 0.328 | 0.656 |
| **GM-CSF** | -0.096 | -0.316; 0.124 | 0.388 | 0.764 | -0.015 | -0.255; 0.225 | 0.904 | 0.973 |
| **HGF** | 0.031 | -0.037; 0.1 | 0.37 | 0.764 | 0.012 | -0.063; 0.087 | 0.749 | 0.973 |
| **IFN-α** | -0.028 | -0.079; 0.023 | 0.276 | 0.764 | -0.034 | -0.089; 0.022 | 0.235 | 0.557 |
| **IFN-γ** | -0.047 | -0.118; 0.024 | 0.191 | 0.764 | -0.061 | -0.139; 0.017 | 0.123 | 0.557 |
| **IL-10** | -0.098 | -0.327; 0.132 | 0.4 | 0.764 | 0.018 | -0.229; 0.266 | 0.884 | 0.973 |
| **IL-12** | -0.029 | -0.06; 0.002 | 0.065 | 0.764 | -0.023 | -0.055; 0.01 | 0.172 | 0.557 |
| **IL-13** | -0.077 | -0.2; 0.046 | 0.221 | 0.764 | -0.102 | -0.237; 0.032 | 0.136 | 0.557 |
| **IL-15** | -0.031 | -0.373; 0.311 | 0.857 | 0.895 | 0.026 | -0.349; 0.401 | 0.892 | 0.973 |
| **IL-1RA** | 0.01 | -0.124; 0.144 | 0.879 | 0.895 | 0.022 | -0.126; 0.17 | 0.768 | 0.973 |
| **IL-2** | 0.026 | -0.088; 0.14 | 0.653 | 0.808 | 0.075 | -0.048; 0.198 | 0.228 | 0.557 |
| **IL-2R** | -0.022 | -0.079; 0.035 | 0.445 | 0.764 | -0.018 | -0.08; 0.045 | 0.579 | 0.973 |
| **IL-4** | 0.043 | -0.055; 0.142 | 0.386 | 0.764 | 0.005 | -0.096; 0.107 | 0.916 | 0.973 |
| **IL-5** | -0.014 | -0.224; 0.196 | 0.895 | 0.895 | 0.009 | -0.22; 0.239 | 0.935 | 0.973 |
| **IL-6** | 0.139 | -0.053; 0.33 | 0.154 | 0.764 | 0.179 | -0.025; 0.383 | 0.085 | 0.557 |
| **IL-8** | -0.046 | -0.15; 0.058 | 0.381 | 0.764 | -0.074 | -0.187; 0.04 | 0.203 | 0.557 |
| **IP-10** | -0.032 | -0.122; 0.058 | 0.485 | 0.764 | -0.015 | -0.111; 0.081 | 0.752 | 0.973 |
| **MCP-1** | -0.022 | -0.099; 0.054 | 0.562 | 0.77 | -0.051 | -0.132; 0.03 | 0.214 | 0.557 |
| **MIG** | -0.095 | -0.377; 0.186 | 0.503 | 0.764 | -0.2 | -0.506; 0.106 | 0.199 | 0.557 |
| **MIP-1α** | -0.014 | -0.057; 0.03 | 0.529 | 0.764 | -0.018 | -0.066; 0.03 | 0.453 | 0.841 |
| **MIP-1β** | 0.009 | -0.07; 0.089 | 0.82 | 0.895 | -0.001 | -0.088; 0.086 | 0.981 | 0.981 |
| **RANTES** | 0.067 | -0.026; 0.16 | 0.157 | 0.764 | 0.058 | -0.044; 0.161 | 0.263 | 0.571 |
| **TNF** | 0.019 | -0.063; 0.1 | 0.652 | 0.808 | -0.009 | -0.098; 0.079 | 0.833 | 0.973 |
| **VEGF** | 0.254 | 0.003; 0.505 | **0.047** | 0.764 | 0.315 | 0.041; 0.59 | **0.025** | 0.557 |

Abbreviations: CI, confidence interval

^a^ P-values were computed through linear regressions using log10-transformed marker concentration as outcome and IgG seropositivity as the predictor variable.

**^b^** P-values were adjusted by multiple testing using a Benjamini-Hochberg approach.
