# Supplementary material for: Immunosuppressive and angiogenic cytokine profile associated with Bartonella bacilliformis infection in post-outbreak and endemic areas of Carrion's disease in Peru
Source: PLoS Negl Trop Dis. 2017 Jun 19;11(6):e0005684. doi: 10.1371/journal.pntd.0005684 (PMC5491314; doi:10.1371/journal.pntd.0005684)
Supplement: S7 Table — (DOCX) [file pntd.0005684.s008.docx]

**S7 Table**. Unadjusted and adjusted analysis of the effect of IgG levels on marker levels.

|  | **Unadjusted model** | | | | **Models adjusted by age and area** | | | |
| --- | --- | --- | --- | --- | --- | --- | --- | --- |
|  | **Coefficient** | **95% CI** | **p-value** ^a^ | **BH ^b^** | **Coefficient** | **95% CI** | **p-value** ^a^ | **BH ^b^** |
| **EGF** | 0.648 | -0.204; 1.499 | 0.135 | 0.755 | 0.196 | -0.737; 1.129 | 0.679 | 0.981 |
| **eotaxin** | 0.314 | 0.092; 0.537 | **0.006** | 0.155 | 0.13 | -0.1; 0.36 | 0.265 | 0.884 |
| **G-CSF** | 0.014 | -0.213; 0.24 | 0.906 | 0.982 | -0.003 | -0.255; 0.249 | 0.983 | 0.983 |
| **GM-CSF** | 0.017 | -0.569; 0.604 | 0.953 | 0.986 | 0.331 | -0.32; 0.982 | 0.317 | 0.884 |
| **HGF** | 0.122 | -0.059; 0.304 | 0.185 | 0.755 | 0.074 | -0.129; 0.277 | 0.471 | 0.937 |
| **IFN-α** | -0.074 | -0.209; 0.061 | 0.283 | 0.808 | -0.089 | -0.24; 0.063 | 0.251 | 0.884 |
| **IFN-γ** | -0.033 | -0.222; 0.156 | 0.731 | 0.863 | -0.055 | -0.267; 0.158 | 0.613 | 0.937 |
| **IL-10** | -0.05 | -0.661; 0.562 | 0.873 | 0.982 | 0.355 | -0.316; 1.026 | 0.298 | 0.884 |
| **IL-12** | -0.04 | -0.123; 0.043 | 0.342 | 0.808 | -0.005 | -0.094; 0.084 | 0.909 | 0.983 |
| **IL-13** | 0.101 | -0.227; 0.43 | 0.543 | 0.822 | 0.104 | -0.265; 0.473 | 0.579 | 0.937 |
| **IL-15** | -0.226 | -1.136; 0.683 | 0.623 | 0.822 | -0.061 | -1.081; 0.958 | 0.905 | 0.983 |
| **IL-1RA** | -0.003 | -0.36; 0.354 | 0.986 | 0.986 | 0.019 | -0.383; 0.421 | 0.924 | 0.983 |
| **IL-2** | 0.12 | -0.184; 0.424 | 0.435 | 0.822 | 0.306 | -0.026; 0.638 | 0.07 | 0.884 |
| **IL-2R** | -0.043 | -0.194; 0.108 | 0.576 | 0.822 | -0.021 | -0.191; 0.148 | 0.805 | 0.983 |
| **IL-4** | 0.079 | -0.184; 0.342 | 0.555 | 0.822 | 0.008 | -0.269; 0.285 | 0.954 | 0.983 |
| **IL-5** | 0.101 | -0.458; 0.66 | 0.722 | 0.863 | 0.241 | -0.383; 0.865 | 0.447 | 0.937 |
| **IL-6** | 0.131 | -0.381; 0.644 | 0.613 | 0.822 | 0.155 | -0.405; 0.714 | 0.586 | 0.937 |
| **IL-8** | 0.201 | -0.074; 0.477 | 0.15 | 0.755 | 0.192 | -0.118; 0.501 | 0.223 | 0.884 |
| **IP-10** | 0.08 | -0.159; 0.32 | 0.508 | 0.822 | 0.189 | -0.07; 0.449 | 0.151 | 0.884 |
| **MCP-1** | 0.131 | -0.072; 0.334 | 0.203 | 0.755 | 0.078 | -0.143; 0.3 | 0.486 | 0.937 |
| **MIG** | 0.248 | -0.5; 0.997 | 0.513 | 0.822 | 0.028 | -0.81; 0.866 | 0.947 | 0.983 |
| **MIP-1α** | -0.028 | -0.144; 0.088 | 0.632 | 0.822 | -0.04 | -0.17; 0.091 | 0.548 | 0.937 |
| **MIP-1β** | 0.114 | -0.096; 0.324 | 0.286 | 0.808 | 0.114 | -0.122; 0.35 | 0.34 | 0.884 |
| **RANTES** | 0.166 | -0.083; 0.414 | 0.189 | 0.755 | 0.139 | -0.14; 0.419 | 0.326 | 0.884 |
| **TNF** | 0.105 | -0.111; 0.322 | 0.337 | 0.808 | 0.035 | -0.206; 0.275 | 0.775 | 0.983 |
| **VEGF** | 0.611 | -0.059; 1.28 | 0.073 | 0.755 | 0.765 | 0.016; 1.515 | **0.045** | 0.884 |

Abbreviations: CI, confidence interval

^a^ P-values were computed through linear regressions using log10-transformed marker concentration as outcome and log10-transformed IgG levels as the predictor variable.

**^b^** P-values were adjusted by multiple testing using a Benjamini-Hochberg approach.
